# Identification of lncRNA-miRNA-mRNA Networks in the Lenticular Nucleus Region of the Brain Contributes to Hepatolenticular Degeneration Pathogenesis and Therapy

**DOI:** 10.1007/s12035-023-03631-1

**Published:** 2023-09-27

**Authors:** Wenjie Hao, Wenming Yang, Yue Yang, Ting Cheng, Taohua Wei, Lulu Tang, Nannan Qian, Yulong Yang, Xiang Li, Hailin Jiang, Meixia Wang

**Affiliations:** 1 Department of Neurology, The First Affiliated Hospital of Anhui University of Chinese Medicine, Hefei, China; 2 Center for Xin’an Medicine and Modernization of Traditional Chinese Medicine of IHM, Anhui University of Chinese Medicine, Hefei, China; 3 Key Laboratory of Xin’an Medicine of the Ministry of Education, Anhui University of Chinese Medicine, Hefei, China; 4 Department of Graduate, Guangzhou University of Chinese Medicine, Guangzhou, China

**Keywords:** Hepatolenticular degeneration, Long non-coding RNA, ceRNA

## Abstract

Long non-coding RNAs (lncRNAs) are a recently discovered group of non-coding RNAs that play a crucial role in the regulation of various human diseases, especially in the study of nervous system diseases which has garnered significant attention. However, there is limited knowledge on the identification and function of lncRNAs in hepatolenticular degeneration (HLD). The objective of this study was to identify novel lncRNAs and determine their involvement in the networks associated with HLD. We conducted a comprehensive analysis of RNA sequencing (RNA-seq) data, reverse transcription-quantitative polymerase chain reaction (RT-qPCR), and computational biology to identify novel lncRNAs and explore their potential mechanisms in HLD. We identified 212 differently expressed lncRNAs, with 98 upregulated and 114 downregulated. Additionally, 32 differently expressed mRNAs were found, with 15 upregulated and 17 downregulated. We obtained a total of 1131 pairs of co-expressed lncRNAs and mRNAs by Pearson correlation test and prediction and annotation of the lncRNA-targeted miRNA-mRNA network. The differential lncRNAs identified in this study were found to be involved in various biological functions and signaling pathways. These include translational initiation, motor learning, locomotors behavior, dioxygenase activity, integral component of postsynaptic membrane, neuroactive ligand-receptor interaction, nuclear factor-kappa B (NF-κB) signaling pathway, cholinergic synapse, sphingolipid signaling pathway, and Parkinson’s disease signaling pathway, as revealed by the Gene Ontology (GO) and Kyoto Encyclopedia of Genes and Genomes (KEGG) pathway analyses. Six lncRNAs, including XR_001782921.1 (*P* < 0.01), XR_ 001780581.1 (*P* < 0.01), ENSMUST_00000207119 (*P* < 0.01), XR_865512.2 (*P* < 0.01), TCONS_00005916 (*P* < 0.01), and TCONS_00020683 (*P* < 0.01), showed significant differences in expression levels between the model group and normal group by RT-qPCR. Among these, four lncRNAs (TCONS_00020683, XR_865512.2, XR_001780581.1, and ENSMUST00000207119) displayed a high degree of conservation. This study provides a unique perspective for the pathogenesis and therapy of HLD by constructing the lncRNA-miRNA-mRNA network. This insight provides a foundation for future exploration in this field.

**Supplementary Information:**

The online version contains supplementary material available at 10.1007/s12035-023-03631-1.

## Introduction

Hepatolenticular degeneration (HLD), commonly referred to as Wilson disease (WD), is an infrequent autosomal recessive neurological disorder resulting from mutations occurring in the ATP7B gene. This disorder is distinguished by a persistent and advancing impairment in copper metabolism [1]. This disease’s prevalence exhibits variation across different countries [2–4]. The prevalence of WD in the United Kingdom (UK) was 15.5/million, with males having a slightly higher prevalence of 16.9/million and females having a slightly lower prevalence of 14.1/million [5]. The overall prevalence of WD in the UK population is estimated to be 1 in 7000. Additionally, it is noteworthy that the proportion of individuals carrying a heterozygous ATP7B mutation, which is associated with WD, is unexpectedly high at approximately 1 in 40 [6]. The principal pathological manifestation of HLD entails the accumulation of excessive copper in various tissues and organs, leading to varying degrees of impairment. The nervous system and liver are the most frequently affected, with the lenticular nucleus being the most commonly impacted region within the nervous system [7, 8]. Research conducted with adolescent populations has indicated that the neurological manifestations of HLD typically emerge during the age range of 10 to 20 years, primarily presenting as tremors, dystonia, and symptoms resembling Parkinson’s disease [9, 10].

Furthermore, long non-coding RNAs (lncRNAs), which are generated as by-products of transcription, were previously regarded as transcripts lacking significant biological functionality [11]. Nevertheless, in the past decade, an increasing number of research has demonstrated the involvement of lncRNAs in the transcriptional processes across various species. Moreover, lncRNAs have received increasing attention as a prospective novel mechanism of biological regulation [12, 13]. Numerous studies have demonstrated that lncRNAs possess the capability to engage with microRNA (miRNA) sites, concurrently contending for endogenous RNAs (ceRNAs), consequently influencing and governing the expression of mRNA and target genes. Furthermore, lncRNAs and mRNAs can mutually occupy miRNA-binding sites, interact with one another, and establish a comprehensive ceRNA network [14]. This phenomenon can contribute to the comprehension of the mechanisms underlying pathogenic genes and transcriptional regulatory networks. Additionally, ceRNAs may participate in the regulation of disease-associated target genes at both transcriptional and post-transcriptional levels [15, 16]. However, the precise role of the majority of lncRNAs across various species remains elusive.

Recent research has demonstrated that a considerable number of lncRNAs possess the ability to modulate gene expression both during transcription and post-transcription, thereby exerting a significant influence on the pathogenesis and progression of neurological disorders [17–19]. Several studies have demonstrated that a significant proportion of lncRNAs play a crucial role as a mediator in the process of brain development [20]. For example, this phenomenon can be observed in the inhibitory effects of lncRNA-GAS5 on the polarization of brain M2 microglia, resulting in an expedited process of demyelination [21]. Additionally, it has been observed that lncRNA-associated ceRNA networks play a crucial role in synaptic plasticity, memory, and regulation of neuroinflammation diseases induced by amyloid-β [22].

However, the pathogenesis of lncRNAs in the lenticular nucleus region of the brain affected by HLD remains unexplored. Given the preferential expression of lncRNAs in the nervous system relative to other organ systems, numerous investigations have concentrated on elucidating their associated neurobiological functions. The establishment and maintenance of neural cell identity in brain development, plasticity, and stress response are among the most prominent functions attributed to lncRNAs [23, 24].

Hence, conducting a comprehensive investigation of the functional role of lncRNAs within the lenticular nucleus region of the brain represents a promising approach towards elucidating the molecular mechanisms that underlie the molecular mechanisms underlying of HLD. Nevertheless, the intricate nature of its pathogenesis and the diverse clinical manifestations pose significant challenges for researchers. Furthermore, the absence of an efficacious therapeutic intervention exacerbates the detrimental impact of this condition on patients’ quality of life and imposes a substantial economic burden on both families and society. Understanding the molecular mechanisms of HLD is of utmost importance due to the observed increase in the number of patients affected by this condition in recent years. This article presents an innovative approach to investigating the pathogenesis of HLD by examining molecular regulation and lncRNA, thereby introducing novel therapeutic targets for treatment. In this study, we conducted RNA sequencing (RNA-seq) analysis on the lenticular nucleus region of the brain to establish the lncRNA expression profile in patients with HLD. We identified differentially expressed lncRNAs (DE-lncRNAs) and investigated their biological functions and molecular mechanisms using bioinformatics analysis. The findings from our current lncRNA expression profile and pathway enrichment analysis have the potential to greatly enhance research on the pathogenesis of HLD and facilitate the identification of novel therapeutic targets.

## Materials and Methods

### Animal Experiments and Sample Collection

The Jackson Laboratory Toxic Milk (TX-j) mouse is a highly suitable model for studying HLD, as it exhibits notable liver and brain injury, as well as an early onset of copper deposition. Consequently, this mouse model has been extensively employed in HLD research [25]. There are a novel autosomal recessive mutant discovered in1987 in the C3H/HeJ animal resource population at Jackson Laboratory in Bar Harbor, Maine. The genetic defect is due to a spontaneous recessive point mutation at position 2135 in exon 8 of the Atp7b gene, resulting in a missense of G712D, with the same mutation gene as HLD patients [26]. Previous research has indicated that TX-j mice exhibit excessive accumulation of copper in the thalamus and pectin during their third month of life. Subsequently, by the age of 12 months, there is an observed elevation in copper concentration within the hippocampus and cerebellum of the striatum, while the concentration of copper in the cerebral cortex remains unaltered during this period [27]. As the deposition of copper persisted, a concomitant increase in the concentration of copper within the cerebral cortex was observed [28]. Upon analysis of their behavior, the TX-j mice exhibited minor abnormalities, such as a predilection for utilizing their forelimbs and displaying clumsiness. The study subjects consisted of TX-j mice and wild-type (WT) mice, both of which shared the same genetic background. TX-j mice, known for their high degree of ATP7B sequence homology (82%) with human HLD, are widely recognized as the most representative animal model due to their comparable physiological, pathological, and clinical characteristics.

TX-j mice were used as the model group, and WT mice were used as the normal group, with 16 mice in each group, 6 of which were selected for RNA sequencing analysis and 10 for RT-qPCR analysis. In the isolation cage, the model and normal groups were given independent oxygen and free access to food and water, the light/dark cycle was 12 h, and the feeding lasted 16 weeks. At the 16th week, the mice experienced dislocation of the cervical vertebra resulting in death, and subsequently, brain lenticular nucleus tissues were obtained. Initially, a portion of the tissues was treated with 4% paraformaldehyde for a duration of 3 h, followed by dehydration using ethanol and xylene, embedding in paraffin, and slicing for subsequent pathological analysis. The remaining portions were securely stored in refrigerated tubes at a temperature of − 80 °C.

### Total RNA Isolation

Library construction and RNA sequencing were carried out by OE Company Shanghai (Oebiotech Biomedical Technology Company, Shanghai, China). Total RNA was extracted using the TRIzol reagent according to the manufacturer’s protocol. RNA purity and quantification were evaluated using the NanoDrop 2000 spectrophotometer (Thermo Fisher Scientific, Waltham, MA, USA). RNA integrity was assessed using the Agilent 2100 Bioanalyzer (Agilent Technologies, Santa Clara, CA, USA). One microgram total RNA of each sample with RIN value above 7 was used for library preparation. Then, the libraries were constructed using TruSeq Stranded Total RNA with Ribo-Zero Gold (Illumina, Cat. No. RS-122-2301) according to the manufacturer’s instructions.

### Annotation and Differential Expression of lncRNAs

The libraries were sequenced on an Illumina Novaseq6000 platform; 150-bp paired-end reads were generated. Raw data (raw reads) of fastq format were firstly processed using the Trimmomatic software [29]. In this step, clean data (clean reads) were obtained by removing reads containing adapter and ploy-N or low quality reads from raw data. Based on the genome alignment results for each sample, the StringTie software applies a flow neural network algorithm to reassemble the transcripts [30].

Sequencing reads were mapped to the mouse genome (GRCm38) using HISAT2 [31]. For mRNAs, FPKM of each gene was calculated using Cufflinks, and the read counts of each gene were obtained by HTSeq-count [32, 33]. Differential expression analysis was performed using the DESeq2 R package [34]. *P* value < 0.05 was set as the threshold for significantly differential expression. For lncRNAs, the transcriptome from each dataset was assembled independently using the Cufflinks 2.0 program [35]. All transcriptomes were pooled and merged to generate a final transcriptome using Cuffmerge (Cufflinks 2.0). All transcripts that overlapped with known mRNAs, other non-coding RNA, and non-lncRNA were discarded. Next, the transcripts longer than 200 bp and the number of exons > 2 were picked out, and the CPC (v 0.9-r2), PLEK (v 1.2), CNCI (v 1.0), and Pfam (v 30) were used to predict transcripts with coding potential [36–38]. The novel predicted lncRNAs were obtained through these processes. The characteristics (including length, type, and number of exons) of lncRNA were analyzed after screening. Then, the novel-predicted lncRNAs and known lncRNAs (from NCBI and Ensemble database) were both used for expression calculation and differential screening.

Bowtie2 and eXpress software were used to calculate the expression abundance of each transcript in each sample by sequence similarity comparison [32]. The FPKM method eliminates the effect of transcript length and sequencing volume difference to calculate the transcript expression [39]. Thus, this calculated transcript expression can reflect high or low expression. The FEELnc software was used to count lncRNA types by the position relationship between lncRNA and known protein-encoded transcripts [40]. Then, lncRNAs were filtered based on the count mean value, and only the lncRNAs in at least one group whose counts mean value was > 2 were selected for the next analysis. DESeq2 software was used to standardize the counts of lncRNAs in each sample (BaseMean value was used to estimate the expression amount) [41] and calculate the multiple of difference. The negative binomial (NB) distribution test was used for the difference significance test, and differential lncRNAs were screened according to the difference multiple and difference significance test results. The default difference filter was *P* < 0.05 for non-biological duplicate samples with a difference multiple > 2.

### Prediction and Annotation of the lncRNA-Targeted miRNA-mRNA Network

Furthermore, miRNAs can reverse-regulate the expression of target genes by inhibiting translation or triggering degradation. On the other hand, lncRNAs can regulate mRNA expression and degradation by competing with limited miRNAs, known as ceRNAs. The ceRNAs can regulate the expression of transcripts by competing with mRNAs for the same miRNA response elements (MREs). Both compete for the binding of miRNAs and regulate each other to form ceRNA networks (ceRNETs).

The miRNA regulatory relationship was predicted using Miranda. The threshold parameter settings: *S* ≥ 150, Δ*G* ≤ − 30 kcal/mol and strict 5′ seed pairing. The ceRNA MuTATE method was used to calculate the score between ceRNA relational pairs [42]. The probability of sharing some miRNAs in the ceRNA relational pairs was calculated using a hypergeometric distribution algorithm. Finally, a ceRNA relational pair with high reliability was obtained. These lncRNA-regulated mRNAs and lncRNAs were evaluated for GO and KEGG enrichment analyses.

### Verification of Sequencing Data Using RT-qPCR

In order to corroborate the findings obtained from RNA-seq analysis, we employed RT-qPCR technique to validate the expression of lncRNAs in the identical sample. First, we generated primers for the identified lncRNAs following the principles of PCR primer design. Primers are listed in Table 3. The lenticular nucleus tissue of mice from both experimental groups served as a template, while β-actin was used as the endogenous control. The relative expression levels of lncRNAs were calculated by the 2^−∆∆Ct^ method to detect the recording level of lncRNA transfer in the calibration samples of the non-treated group.

### Statistical Analyses

The mean ± standard deviation (SD) was used to express the quantitative data obtained from RT-qPCR. Student’s *t*-test was conducted using the Statistical software package for Social Science 22.0 (SPSS, Chicago, USA). A significance level of *P* < 0.05 was deemed statistically significant.

## Results

### Mice Genotyping and Lenticular Nucleus Histopathology

**Fig. 1:**
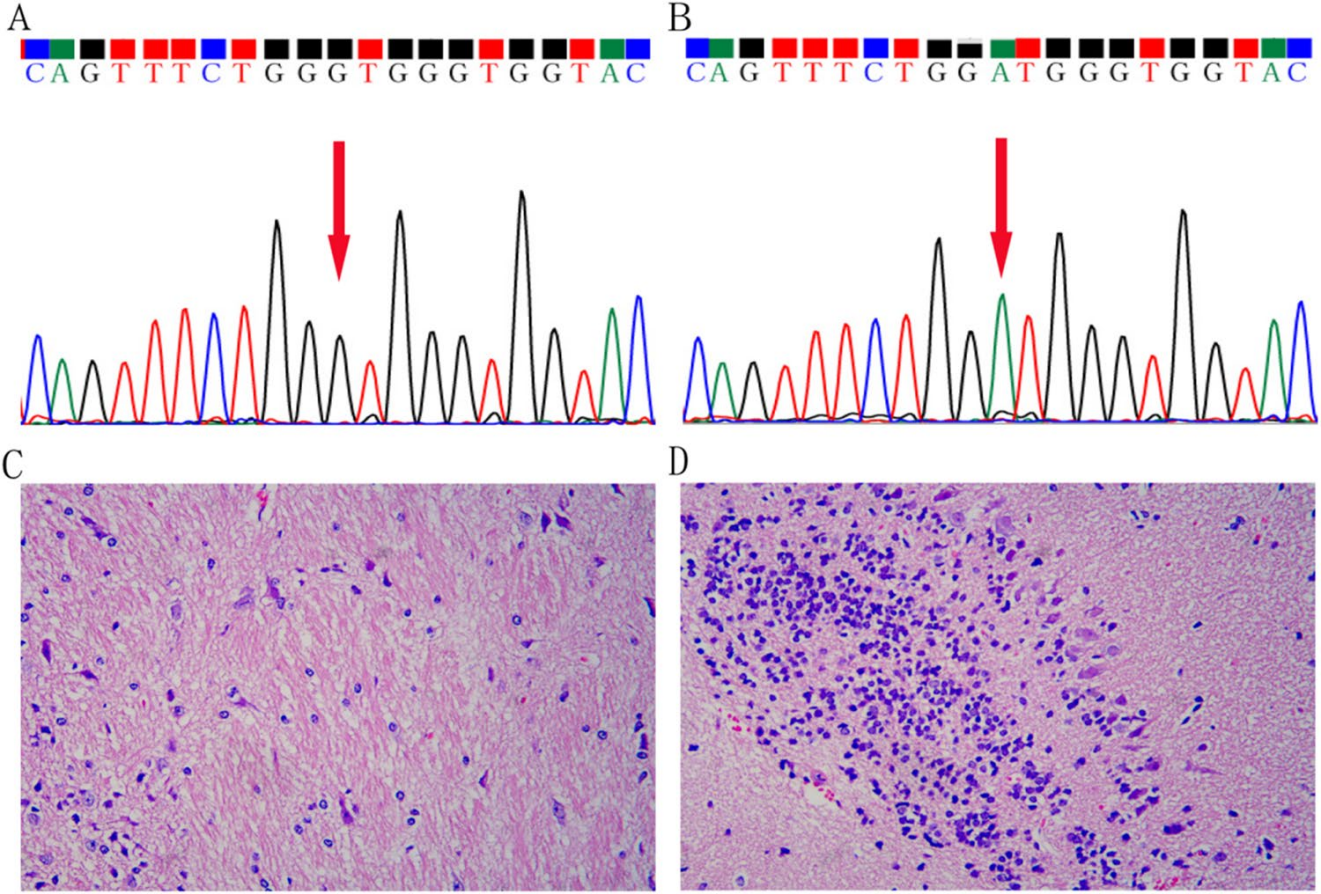
The genotyping of mice and the histopathological examination of the lenticular nucleus. **A** The mice in the normal group had a diploid wild-type genotype; **B** the mice in the model group had a diploid mutant genotype. Lenticular nucleus histopathology in the **C** normal and **D** model groups (× 400)

First, all mice were genotyped as diploid mutant Jackson Laboratory Toxic Milk mice (TX-j) (model group, MOD) or diploid wild-type mice (normal group, NOR) (Fig. 1A, B). The hematoxylin and eosin staining showed that a large number of inflammatory cells were infiltrated, some neurons were shrunken, and nerve fibers were loose in the lenticular nucleus region of the model group compared to the normal group (Fig. 1C, D).

### Summary of the RNA-seq Data

Total transcriptome sequencing was performed on the brain’s lenticular nucleus tissue of three model mice (TX-j mice) and three normal mice, resulting in a total of 83.1 G of clean data. The effective data amount of each sample ranged from 13.34 to 14.41 G, the Q30 base was distributed in 95.02–95.12%, and the average GC content was 47.99%. Reads were compared to the reference genome to obtain the genome alignments of each sample. The alignment rate was 96.58–96.72%. Overall, these results indicated that the sequencing data were sufficiently representative and valid.

### Expression Profile of lncRNAs in the Lenticular Nucleus

**Fig. 2:**
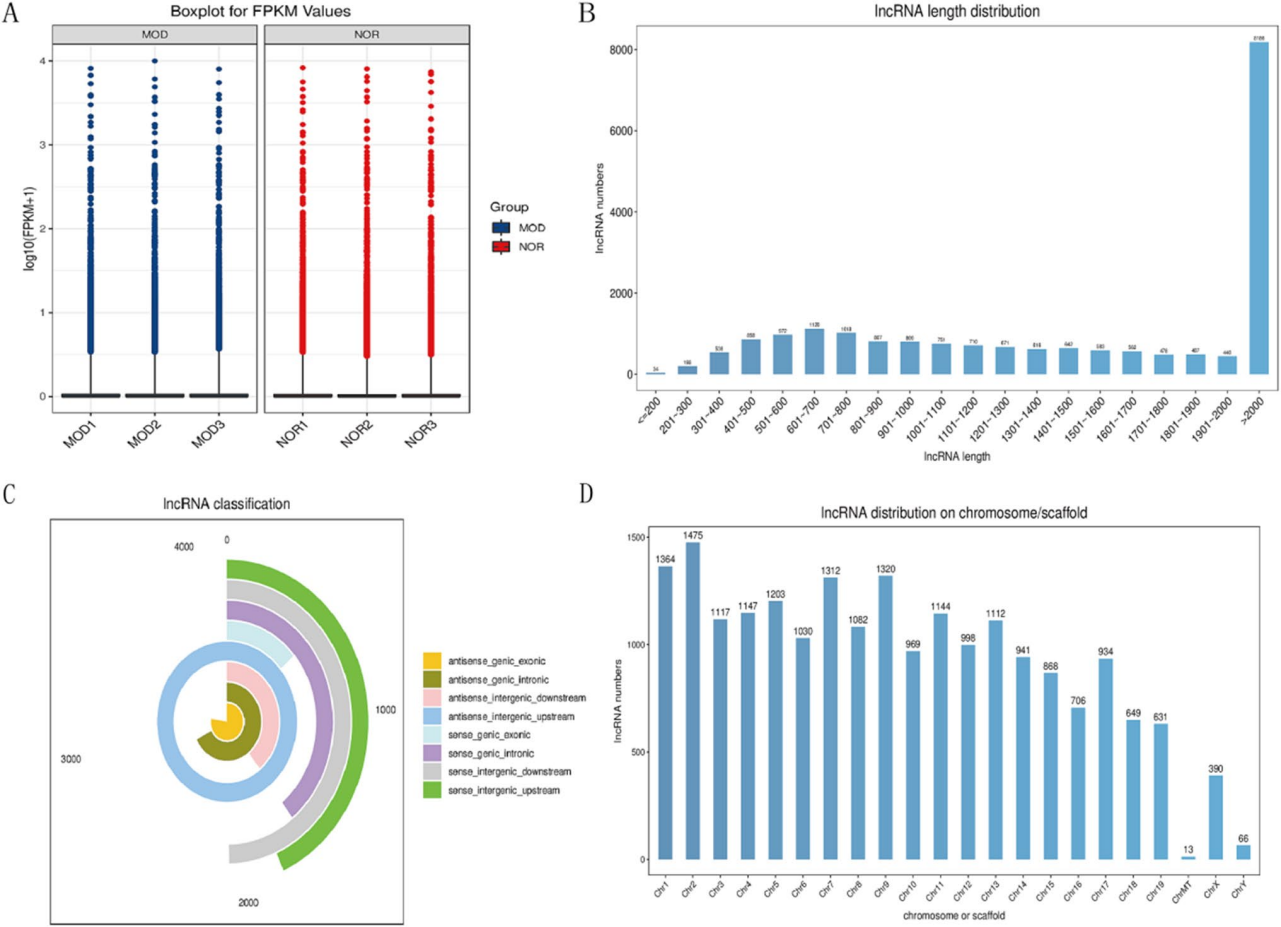
Expression profile of lncRNAs in the lenticular nucleus. **A** FPKM value of lncRNA expression in each sample; **B** lncRNA sequence length distribution range; **C** lncRNA types; **D** distribution of lncRNAs on chromosomes

The lenticular nucleus expression patterns of three model mice (TX-j mice) and three normal mice were studied using deep RNA sequencing. In the three model samples and three normal samples, a total of 20,471 lncRNA transcripts were identified, and all FPKM values of lncRNA expression were > 0 (Fig. 2A). The length of lncRNA sequences ranged from 73~93147 nt, and the sequences > 2000 nt accounted for the largest proportion (39.99%) (Fig. 2B). The most common lncRNA type was antisense-intergenic-upstream followed by downstream antisense-genic-exonic, antisense-genic-intronic, sense-intergenic-downstream, and sense-genic-exonic (Fig. 2C). Furthermore, we evaluated the distribution of lncRNAs on mouse chromosomes and identified 20,471 lncRNA transcripts on all chromosomes, including Chr X and Chr Y, with Chr 2 having the most lncRNAs (Fig. 2D).

### Differentially Expressed lncRNAs (DE-lncRNAs) and mRNAs(DE-mRNAs)

**Fig. 3:**
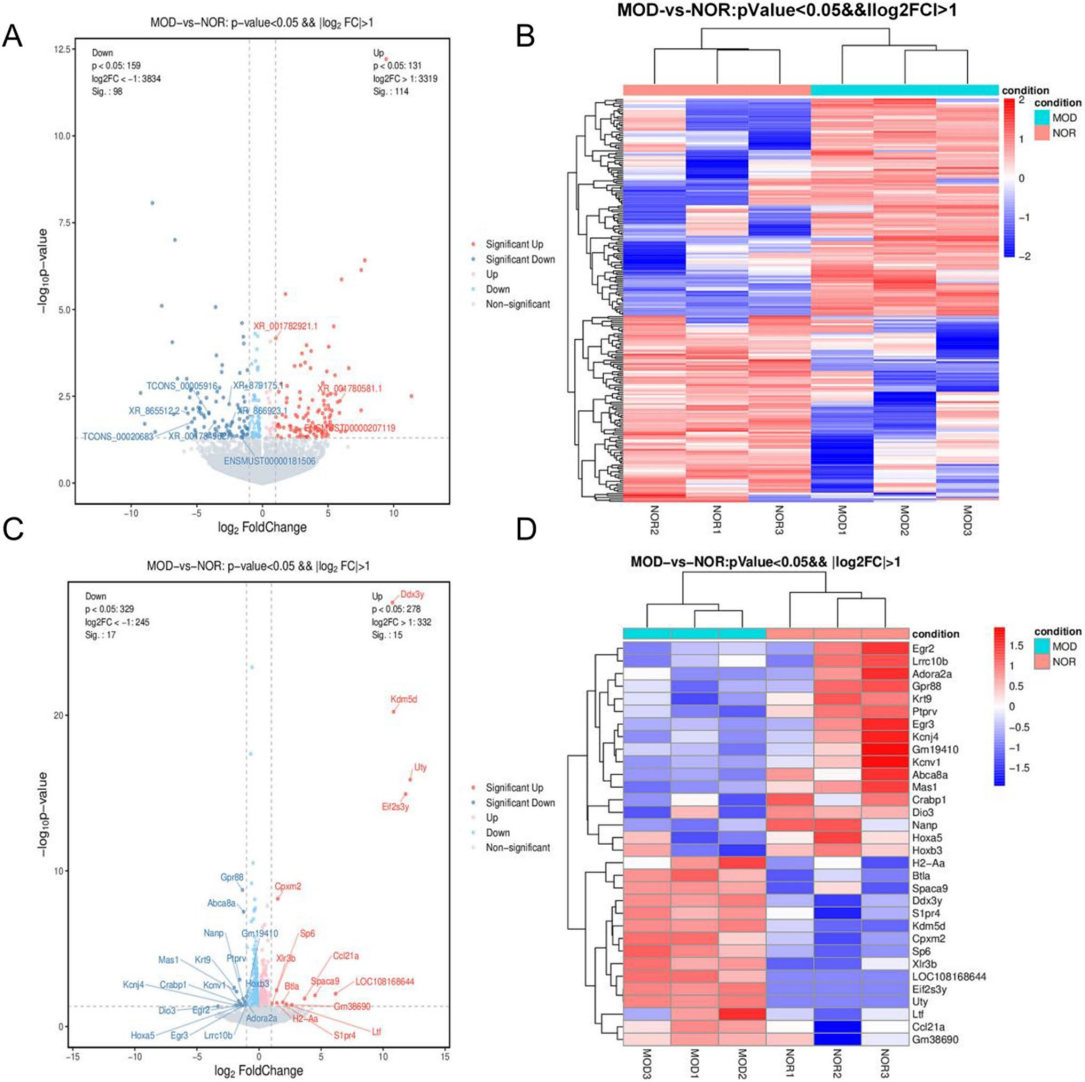
Differentially expressed lncRNAs (NOR vs. MOD) in the lenticular nucleus as compared to normal control. **A** Volcano map of DE-lncRNAs; **B** heat map of DE-lncRNAs; **C** volcano map of DE-mRNAs; **D** heat map of DE-mRNAs

In order to investigate the involvement of lncRNAs and mRNAs in the lenticular nucleus of mice with HLD, we conducted a comparative analysis of lncRNA expression levels between the lenticular nucleus regions of the model and normal groups. Subsequently, we identified DE-lncRNAs based on the following criteria: a Log2|fold change (FC)| ≥ 1 and corrected *P* values < 0.05. A total of 212 DE-lncRNAs were detected, 98 upregulated and 114 downregulated. A total of 32 DE-mRNAs were detected, 15 upregulated and 17 downregulated. Volcano and heat maps are used to show the DE-lncRNAs and DE-mRNAs (Fig. 3).

### Co-expression of DE-lncRNAs and mRNAs

**Fig. 4:**
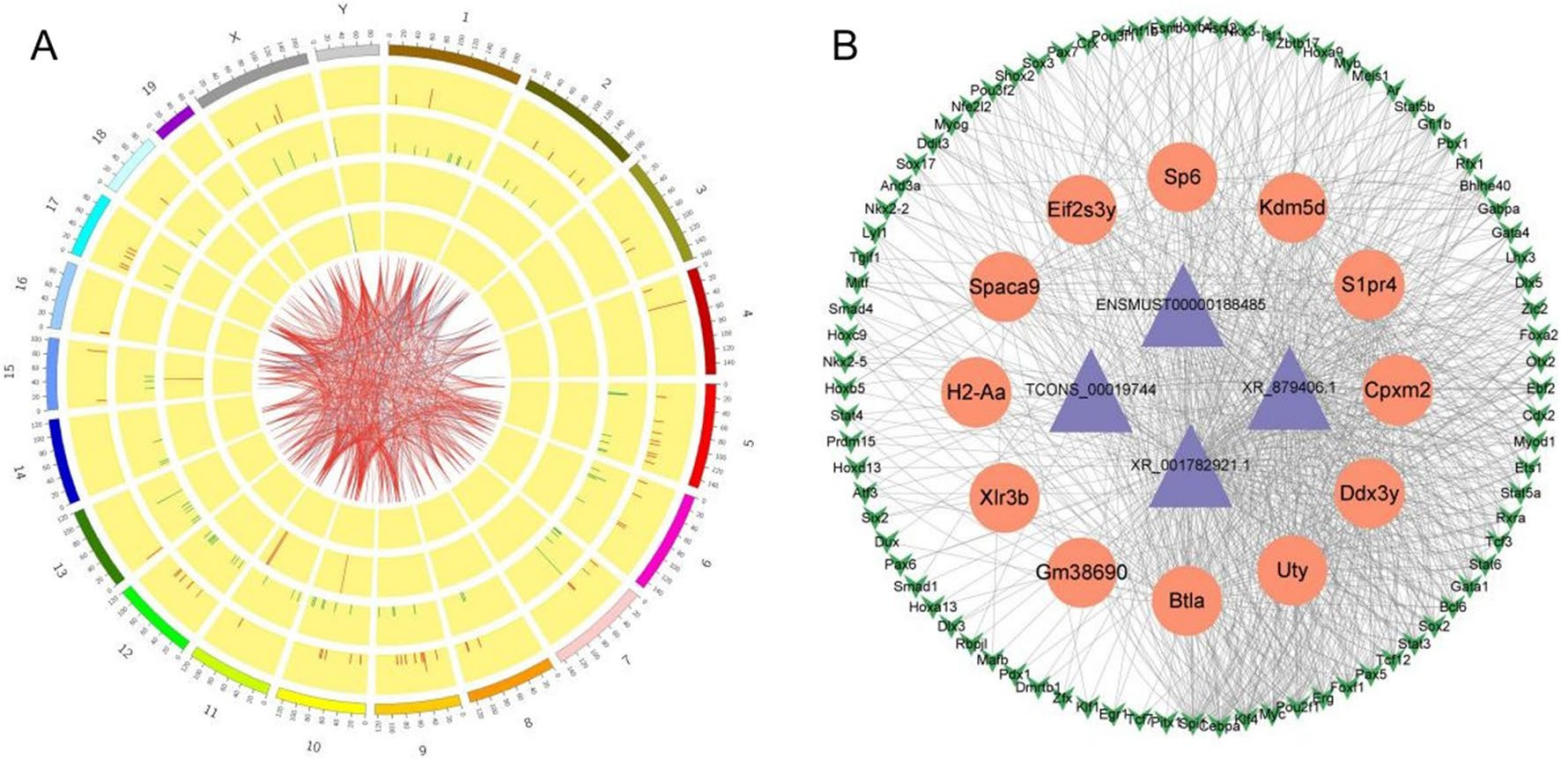
**A** Circos graph (this graph only represents the distribution of DE-mRNAs/DE-lncRNAs on the chromosome, not the number). The outermost circle represents the autosomal distribution diagram of this species. In the second and third circles, differentially expressed mRNAs are distributed on chromosomes. The red line denotes upregulation, whereas the green line denotes downregulation. The greater the bar, the more mRNA was differently expressed at this location. The fourth and fifth circles show the distribution of DE-lncRNAs on chromosomes, expressed in the same form as mRNAs. The internal line indicates the mapping between the top 500 co-expressed lncRNAs and mRNAs. **B** Ternary regulation network of lncRNA-TF-mRNA. The blue nodes represent lncRNAs, the orange nodes mRNAs, and the green nodes TFs

The Pearson correlation test was used to calculate the expression correlation between DE-lncRNAs (length < 6000 nt) and differential mRNA expression data. The threshold was a correlation coefficient ≥ 0.8 and a *P* < 0.05. Finally, a total of 1131 pairs of co-expressed genes were obtained. To more intuitively display this information, DE-lncRNAs and mRNAs in the same comparison group were mapped using a circos graph [43] (Fig. 4A). A table listing the top 500 co-expressed lncRNAs and mRNAs with correlation coefficient and *P* values as Supplementary Information (Supplementary table 1).

### Association of lncRNAs with Transcription Factors (TFs)

Furthermore, lncRNAs that possess the ability to interact with TFs actively engage in regulatory processes by recruiting TFs and guiding them to specific regions within the DNA sequence, such as the promoter region, thereby exerting control over transcriptional activity. Additionally, an alternative regulatory mechanism involves the binding of multiple TFs to lncRNA molecules. In instances where multiple signaling pathways are concurrently activated within an organism, these downstream effector molecules can associate with the same lncRNA, facilitating the convergence and integration of information across distinct signaling pathways.

Based on the co-expression of lncRNAs and mRNAs, TF potential binding of lncRNAs was predicted using TF data on the JASPAR database [44]. The GTRD database’s gene-transcription factor pairings and lncRNA-mRNA co-expression were used to build the three-element regulatory network of lncRNA-TF-mRNA [45], extract the top 500 relationship pairs, and draw the ternary regulatory network diagram using a network software package [46] (Fig. 4B). Finally, we obtained four major lncRNAs (ENSMUST00000188485, TCONS_00019744, XR_001782921.1, and XR_879406.1) that were involved in the regulation of twelve mRNAs (Sp6, Kdm5d, S1pr4, Cpxm2, Ddx3y, Uty, Btla, Gm38690, X1r3b, H2-Aa, Spaca8, and Eif2s3y) by binding to 51 major TFs.

### Prediction and Annotation of the lncRNA-Targeted miRNA-mRNA Network

The regulation and degradation of mRNAs by lncRNAs compete with limited miRNAs, known as competing for endogenous RNAs (ceRNAs). These ceRNAs can compete with other RNA transcripts for the same miRNA, to achieve mutual communication and regulation, including protein-coding genes, pseudogenes, and lncRNAs [47]. The ceRNA hypothesis is based on research on how RNA transcripts interact with each other. Additionally, miRNAs are 22 nt short RNAs that can reverse-regulate target gene expression by inhibiting translation or degradation. The ceRNA hypothesis suggests that ceRNA regulates the expression of transcripts by competing for the same MREs as mRNAs. Regardless of their protein-encoding capacity, RNA transcripts can compete with each other to bind to miRNAs and also regulate each other to form huge ceRNA networks (ceRNETs).

Herein, lenticular nucleus tissues of three model mice and three normal mice were analyzed, and the total number of differential lncRNAs, miRNAs, and mRNAs was 212, 1963, and 32, respectively. The Miranda program was used to predict the binding between miRNA-mRNA and miRNA-lncRNA sequences. The parameters were set as the Miranda default V3.3a. A total of 1135 miRNA-mRNA pairs and 3731 miRNA-lncRNA pairs were finally obtained. The top 20 pairs with the highest expression correlation were selected and displayed (Tables 1 and 2).

**Table 1:** First 20 relationship pairs of miRNA-lncRNA co-expression and target gene prediction diagram

miRNA	lncRNA	Total score	Total energy	Max score	Max energy	Target length	MRE
mmu-miR-466i-3p	XR_001780581.1	1258	− 247.08	196	− 37.37	23199	7
mmu-miR-466m-3p	XR_001780581.1	1248	− 258.44	196	− 38.23	23199	7
mmu-miR-466f-3p	XR_001780581.1	1237	− 258.04	191	− 38.86	23199	7
mmu-miR-466b-3p>mmu-miR-466c-3p>mmu-miR-466p-3p	XR_001780581.1	1126	− 220.86	171	− 36	23199	7
mmu-miR-466h-3p	XR_001780581.1	1071	− 235.25	153	− 35.08	23199	7
mmu-miR-877-3p	ENSMUST00000232598	1008	− 196.71	172	− 35.19	1890	6
mmu-miR-669f-3p	XR_001780581.1	1002	− 215.96	177	− 37.92	23199	6
mmu-miR-466a-3p>mmu-miR-466e-3p	XR_001780581.1	1001	− 200.5	176	− 37.71	23199	6
mmu-miR-3960	ENSMUST00000057889	993	− 231.42	176	− 48.15	2248	6
mmu-miR-669b-3p	XR_001780581.1	978	− 185.83	173	− 31.88	23199	6
mmu-miR-466d-3p	XR_001780581.1	966	− 193.38	171	− 33.03	23199	6
mmu-miR-7687-5p	ENSMUST00000200143	948	− 198.89	163	− 36.61	1460	6
mmu-miR-466i-5p	XR_382195.2	898	− 180	190	− 38.3	5114	5
mmu-miR-7058-3p	TCONS_00006180	816	− 161.02	168	− 35.11	2772	5
mmu-miR-5126	ENSMUST00000200143	808	− 201.19	170	− 45.88	1460	5
mmu-miR-467a-3p	XR_001780581.1	803	− 163.34	168	− 34.32	23199	5
mmu-miR-877-3p	ENSMUST00000185568	799	− 162.04	163	− 32.99	2380	5
mmu-miR-877-3p	NR_015605.1	799	− 162.04	163	− 32.99	2585	5
mmu-miR-467f	XR_001780581.1	798	− 154.19	167	− 32.49	23199	5
mmu-miR-7081-5p	NR_038048.1	793	− 164.09	165	− 35.99	2677	5

**Table 2:** Co-expression of miRNA-mRNA and prediction of target genes in the first 20 relationship pairs

miRNA	mRNA	Total score	Total energy	Max score	Max energy	Target length	MRE
mmu-miR-5107-5p	LOC108168644	3986	− 817.61	167	− 33.01	5689	26
mmu-miR-346-3p	LOC108168644	3638	− 741.37	155	− 32.86	5689	24
mmu-miR-504-3p	LOC108168644	3051	− 672.44	161	− 36.04	5689	19
mmu-miR-7222-3p	LOC108168644	2898	− 564.08	175	− 33.45	5689	17
mmu-miR-1249-5p	LOC108168644	2632	− 543.3	184	− 40.4	5689	17
mmu-miR-3572-5p	LOC108168644	2567	− 531.59	151	− 31.27	5689	17
mmu-miR-6987-5p	LOC108168644	2287	− 464.1	184	− 39.74	5689	13
mmu-miR-5110	LOC108168644	2097	− 413.13	172	− 34.79	5689	13
mmu-miR-7118-5p	LOC108168644	1459	− 293.17	168	− 33.44	5689	9
mmu-miR-207	Krt9	1082	− 222.87	157	− 35.52	2580	7
mmu-miR-1893	LOC108168644	930	− 182.22	155	− 30.37	5689	6
mmu-miR-7011-3p	Krt9	930	− 206.35	161	− 35.92	2580	6
mmu-miR-149-3p	Hoxb3	812	− 182.24	171	− 41.69	8699	5
mmu-miR-149-3p	LOC108168644	811	− 185.38	166	− 39.72	5689	5
mmu-miR-7081-5p	Kcnj4	790	− 158.48	171	− 34.11	6565	5
mmu-miR-328-5p	LOC108168644	750	− 182.55	150	− 36.51	5689	5
mmu-miR-466i-3p	Kcnj4	720	− 142.74	180	− 35.79	6565	4
mmu-miR-466m-3p	Kcnj4	712	− 153.18	180	− 40.05	6565	4
mmu-miR-466f-3p	Kcnj4	700	− 147.94	175	− 38.83	6565	4
mmu-miR-1249-5p	Hoxb3	679	− 135.99	176	− 36.45	8699	4

**Fig. 5:**
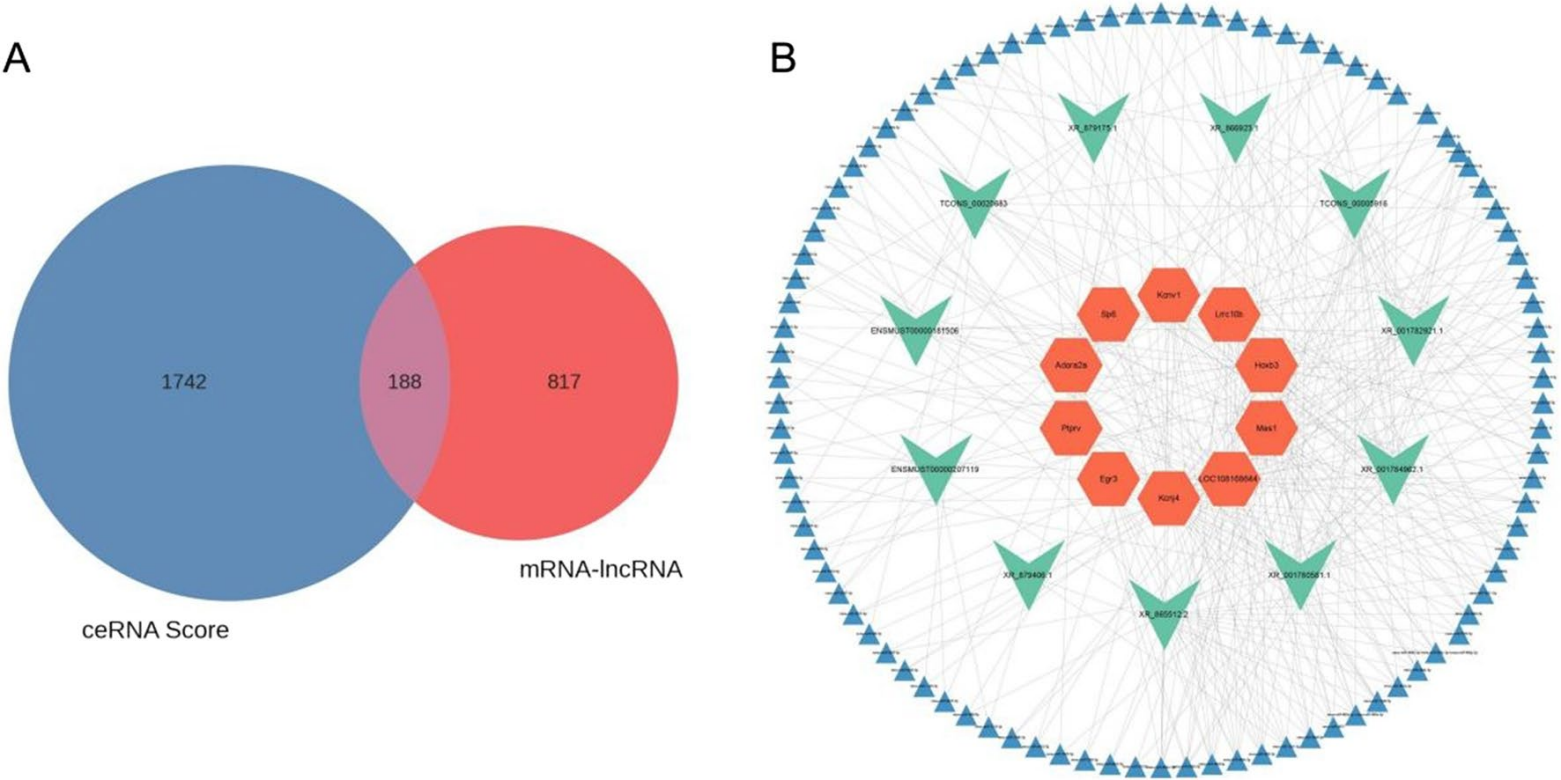
**A** Venn diagram of the relationship between ceRNAs and mRNA-lncRNA pairs. The calculation results of mRNA and lncRNA co-expression were used to filter the ceRNA score results. **B** ceRNA ternary network diagram. The orange nodes represent mRNAs, cyan nodes represent lncRNAs, and blue nodes represent miRNAs

For the relationship pairs with regulatory roles, the ceRNA score was calculated based on the MuTaME method [48]. The *P* value for the matching ceRNA relationship was simultaneously determined using a combined hypergeometric distribution. The smaller the *P* value, the more significant these miRNAs were shared between two ceRNAs (mRNA and Target). A total of 817 mRNA-lncRNA pairs with positive correlation were evaluated based on their inceRNA function associations (Fig. 5A).

Among the top 100 mRNA-lncRNA relationship pairs with the highest MuTATE score in the ceRNA analysis results, the ternary network diagram of ceRNA was drawn for 200 mRNA-miRNA-lncRNA relationship pairs (Fig. 5B). The ceRNA ternary relation network of lncRNAs included XR_879175.1, XR_866923.1, TCONS_00005916, XR_001782921.1, XR_001784962.1, XR_001780581.1, XR_ 865512.2, XR_879406.1, ENSMUST00000207119, ENSMUST00000181506, and TCONS_00020683. The mRNAs included Kcnj4, LOC108168644, Kcnv1, Sp6, Mas1, Lrrc10b, Egr3, Adora2a, Ptprv, and Hoxb3.

**Fig. 6:**
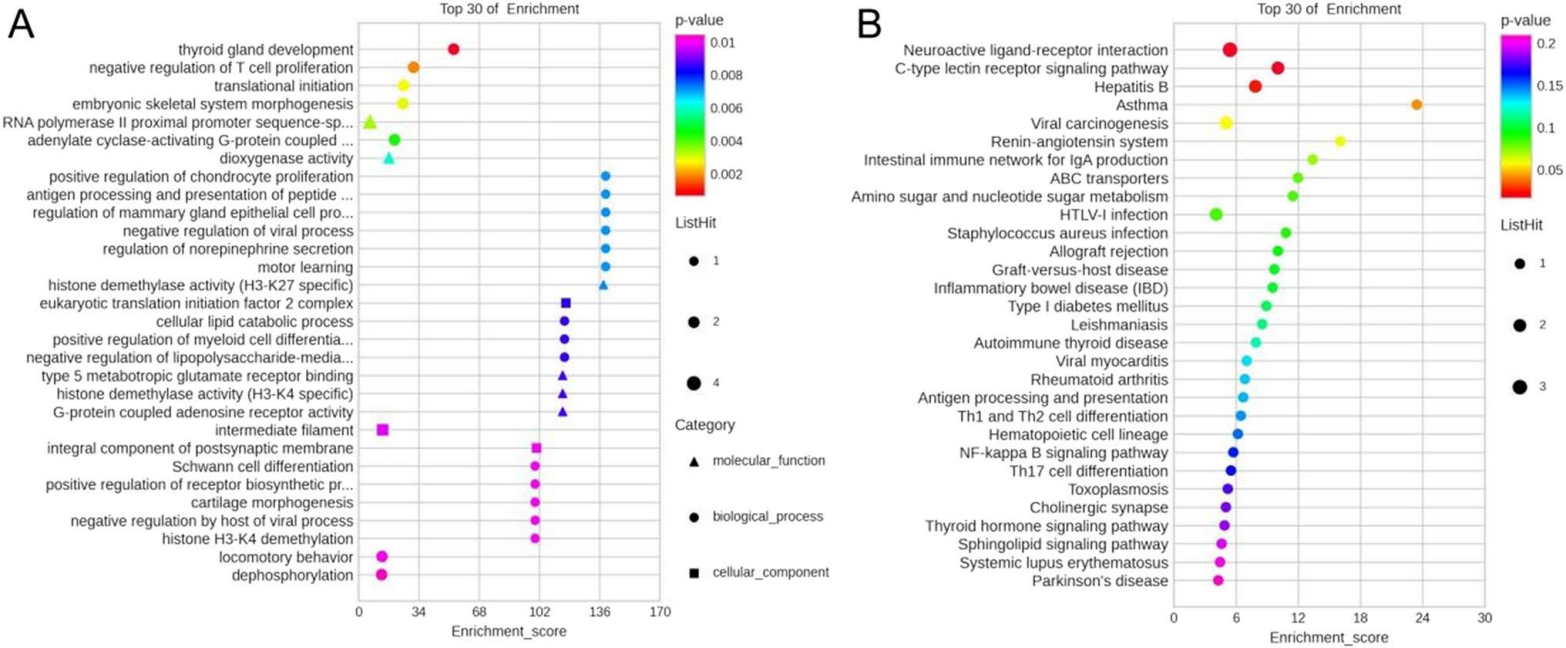
Enrichment analysis of protein-coding genes in the entire lncRNA-mRNA co-expression network by GO and KEGG. The top 30 items are presented. **A** GO and **B** KEGG bubble maps

Since lncRNAs and mRNAs communicate through shared miRNAs, they can also present similar functions. A hypergeometric distribution test was used to calculate the GO and KEGG enrichment significance of these mRNAs regulated by lncRNAs and comprehended the functional annotation of these DE-lncRNAs [49]. Protein coding genes in the entire lncRNA-mRNA co-expression network were analyzed, and the first 30 items enriched by GO and KEGG are presented in Fig. 6A, B, respectively. According to the GO enrichment analysis, DE-lncRNAs were mostly enriched for biological processes such as learning locomotory behavior of the translational initiation motor. The RNA polymerase II proximal promoter sequence-specific DNA binding, dioxygenase activity, and type 5 metabotropic glutamate receptor binding function were the molecular functions involved. The integral component of the postsynaptic membrane was the cellular component enriched. The neuroactive ligand-receptor interaction pathway was the primary enrichment of lncRNAs according to the KEGG enrichment analysis (the highest number and the lowest *P* value). Additionally, ABC transporters, amino sugar and nucleotide sugar metabolism, NF-κB signaling pathway, cholinergic synapse, sphingolipid signaling pathway, and Parkinson’s disease pathway were closely related.

### Validation by RT-qPCR

**Fig. 7:**
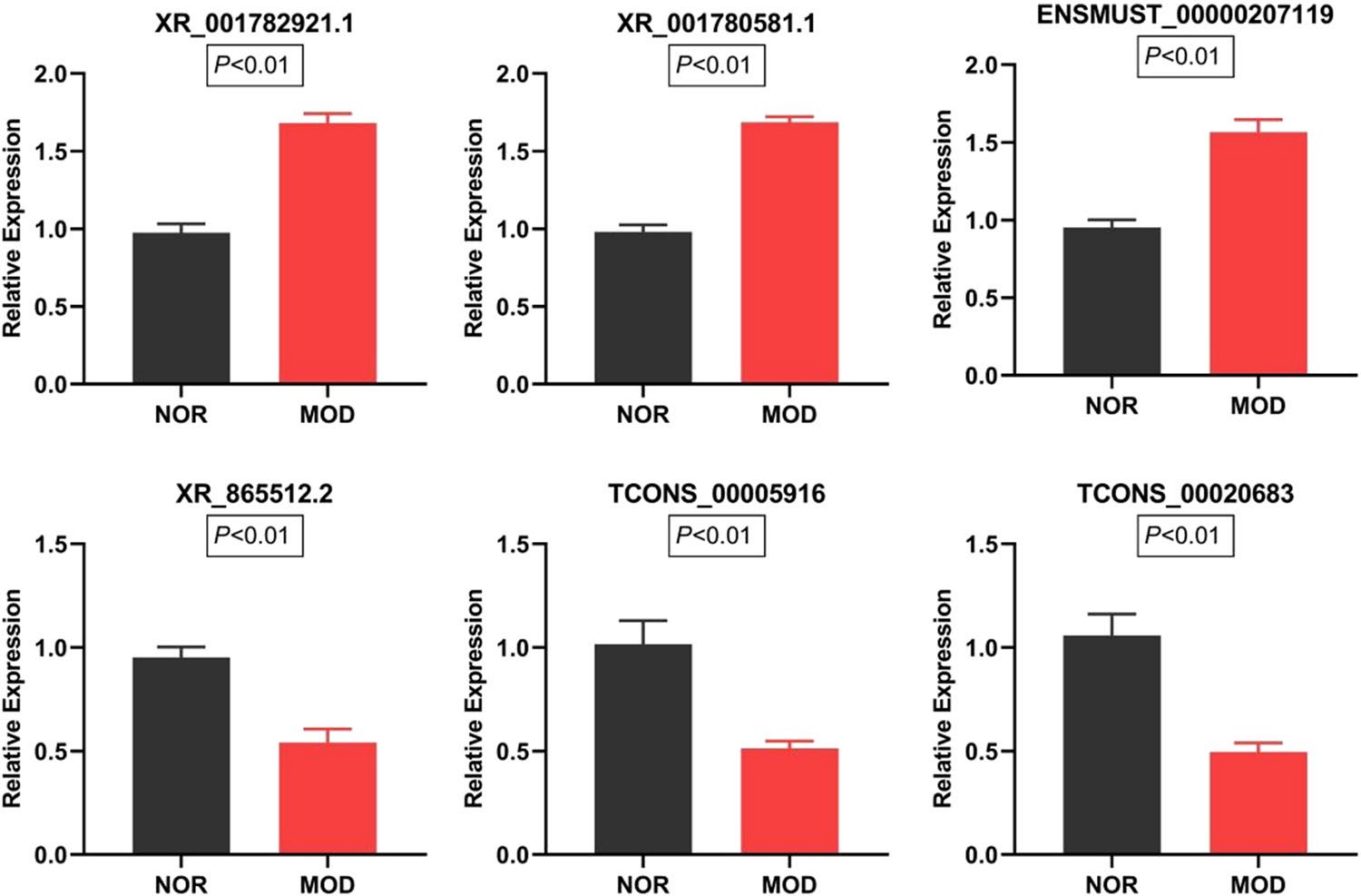
RT-qPCR validation of the selected DE-lncRNAs

Eleven DE-lncRNAs in the ceRNA ternary relational network were selected for RT-qPCR detection and verification, and we found that 6 of them had significant differences in expression (Fig. 7), indicating the reliability of the sequencing analysis results. Primers are listed in Table 3.

**Table 3:** Primers for RT-qPCR validation

lncRNA_id	Primers
XR_001782921.1	Forward: TTCACACTGAGAGGGTTGCT
	Reverse: GAGTTTCCTGGGTCCTGGTT
XR_001780581.1	Forward: CGTGACATCCGACGAACAAA
	Reverse: TAGGATGCAGAGCCCAACAA
ENSMUST_00000207119	Forward: CCTGTGGCCTTAAAGATGCC
	Reverse: TGTGTCACACCCACCTCTAC
XR_865512.2	Forward: AGCTCACGCAAGTTTGTTGT
	Reverse: GTCCCAAGATGCACAAGTGG
TCONS_00005916	Forward: TCACCCCTAGCAGAGTGGTA
	Reverse: GTTCTGGGATGGGGAGTTCA
TCONS_00020683	Forward: TACCTGTATACCTTTGTTTG
	Reverse: GCTGGAAAATAGGATGAAAT

### Conservation Analysis of lncRNAs

Eleven DE-lncRNAs in ceRNA ternary network were selected for comparison with human blast. Through conservation analysis, the human orthologous lncRNA of 5 murine lncRNAs were obtained, indicating that these 5 lncRNAs are highly conserved (Table 4). Combined with the results of RT-qPCR, we found that these 4 lncRNAs (TCONS_00020683, XR_865512.2, XR_001780581.1, ENSMUST00000207119) not only had high conservation, but also had obvious expression differences. We will carry out further research and analysis on these 4 lncRNAs, such as lncRNA function analysis, or comparison with lncRNA expression profile of HLD patients.

**Table 4:** Conservation analysis of lncRNAs

Query id	Subject id	% identity	*e*-value
TCONS_00020683	ENST00000565467	97.5	4.00E−11
XR_865512.2	NR_002813.1	100	1.00E−09
XR_879175.1	ENST00000564508	83.77	3.00E−17
XR_001780581.1	XR_942049.2	96.36	1.00E−16
ENSMUST00000207119	ENST00000605834	91.07	2.00E−08

## Discussion

Previously, HLD was widely perceived as an infrequent ailment, resulting in limited scholarly investigations. Presently, HLD has become more prevalent, imposing a greater economic strain on both society and families. Consequently, research endeavors pertaining to HLD have progressively intensified, albeit the comprehensive understanding of its pathogenic molecular mechanism remains elusive. Furthermore, the prominence of lncRNAs has surged in recent years due to their involvement in diverse biological processes [50]. However, their association with HLD remains unknown. Hence, in this study, we used high-throughput transcriptome sequencing to evaluate the expression of lncRNAs in the lenticular nucleus region of HLD. Through the co-expression correlation analysis of DE-lncRNA and DE-mRNA, the relationship between lncRNA and transcription factors, and ceRNA network analysis, the lncRNA regulatory network was obtained, and 11 lncRNAs with very important roles in the regulatory network were screened out. Furthermore, four important lncRNAs were identified by lncRNA conservation analysis and RT-qPCR validation. Further functional analysis of these four lncRNAs will be performed in the future. These results contribute to the understanding of the complex neurological pathogenesis of HLD. To the best of our knowledge, this is the first comprehensive transcriptome analysis of lncRNA expression profiles in the lenticular nucleus region of HLD.

In this study, lncRNA transcription factor association analysis was employed to assess the functional role of DE-lncRNAs. The findings revealed the involvement of four primary lncRNAs in the regulation of six mRNAs through the binding of 51 key transcription factors. Furthermore, we performed a comprehensive analysis of specific lncRNAs transcription factors and subsequently developed a network model of lncRNA-miRNA-mRNA interactions, known as ceRNA network, which included 10 lncRNAs, 92 miRNAs, and 10 mRNAs.

Among these lncRNAs, namely, TCONS_00020683, XR_865512.2, XR_001780581.1, and ENSMUST00000207119, it is evident that they exhibit higher conservation levels, suggesting their potential significance in the pathogenesis of HLD. Burkhead JL’s studies on the mouse model for HLD (Atp7b(-)(/-)) revealed copper accumulation in hepatic nuclei and specific changes in mRNA profile prior to the onset of pathology. They found that remodeling of the RNA processing machinery is an important component of cell response to elevated copper that may guide pathology development in the early stages of HLD [51]. In the past, our research group has done a lot of research using RNA-seq to explore the pathogenesis of liver and kidney injury in HLD. We elucidated the lncRNA-mRNA regulation network in HLD liver injury in a TX-j WD mouse model using RNA sequencing, and constructed differential lncRNA and mRNA co-expression networks. The identified differential lncRNAs involved in the pathogenesis and development of HLD liver injury [52]. Then, we performed gene expression profiling of Gan-Dou-Fu-Mu decoction (GDFMD)-treated TX-j WD model mice using RNA-Seq analysis and found the genes, pathways, and processes effected by the treatment. Our study provides a theoretical basis to prevent liver fibrosis resulting from WD using GDFMD [53]. Moreover, we identified the circRNA/miRNA/mRNA network involved in kidney failure in HLD, which may serve as a potential biomarker for the pathogenesis of HLD [54].

This study additionally identified Sp6, Mas1, Egr3, Adora2a, and Hoxb3 mRNA as potential significant biomarkers for HLD. The Sp6 gene is implicated in the proliferation of cells during early development, primarily exhibiting expression within the developing germ layer. Subsequently, it undergoes differentiation into enamel and epidermis tissues within the nervous system [55, 56]. The recent association of Sp6 expression in the amygdala and hippocampus with cognitive function and motor disability-related diseases has been established [57]. Moreover, the Sp6 gene has been linked to the formation of a crucial anatomical structure in the eye known as the optical (pigment) cup [58]. These results indicated that Sp6 may play a role in the pathogenesis of HLD dyskinesia and corneal K-F ring symptoms. The MAS receptor, encoded by the mas1 gene, is observed in microglia and primarily associated with the renin-angiotensin system and the stimulation of anti-inflammatory signaling pathways [59, 60]. Microglia have been extensively studied in the context of brain injury as central nervous system guardians [61]. Copper ions deposited in the brain can cause inflammation and damage microglia. Mas1 regulates the NF-κB signaling pathway that plays an important role in the development and pathology of the nervous system. The role of NF-κB activity in the nervous system is usually based on the control of neuronal apoptosis, neurite growth, and synaptic plasticity [62, 63]. From this we can know, Mas1 may potentially be associated with the inflammatory response of HLD and consequently contribute to the development of its pathogenesis. Egr3 is essential for nervous system development, particularly in the sympathetic autonomic nervous system [64]. Egr3 is also involved in motor coordination and motor skill learning functions [65]. The Adora2a gene’s polymorphism has been found to be linked to the occurrence of central nervous system disorders, including attention-deficit hyperactivity disorder (ADHD) and Tourette’s syndrome [66, 67]. Additionally, in the central nervous system, the activation of Adora2a can lead to neuronal damage, while also augmenting excitatory neurotransmitters and potentially causing damage to the white matter [68–70]. White matter injury is frequently observed in patients with HLD from a clinical perspective. Hoxb3 is a prominent member of the Hoxbs family that is expressed in neural stem cells at a young age. It regulates neuron development and proliferation and is involved in nerve cell differentiation and oligodendrocyte progenitor cell apoptosis in microglia [71, 72]. The interrelated functions of these mRNAs exhibit close associations with the pathological and clinical manifestations observed in HLD. In subsequent investigations, my research will primarily concentrate on elucidating the precise mechanisms underlying the involvement of these mRNAs in HLD.

The GO analysis revealed that the primary biological functions implicated were motor learning, locomotor behavior, and dioxygenase activity. The neurological manifestations of HLD primarily consist of extrapyramidal symptoms. Dysphasia (dyskinesia) is the predominant initial presentation, observed in 85–97% of cases [73]. Additional common neurological manifestations encompass bradykinesia, facial grimacing, dystonia, tremor, rigidity (characterized by a lead pipe rather than buckling or gear pattern), urinary incontinence, hyperreflexia, and other symptoms [74]. Furthermore, cognitive impairment may be present in certain patients [75]. The biological functions identified through GO analysis exhibit a strong correlation with the clinical attributes of HLD, thus warranting further investigation into the lncRNAs and mRNAs associated with these biological functions.

The KEGG analysis revealed significant enrichment of the NF-κB, cholinergic synapse, sphingolipid, and Parkinson’s disease signaling pathways. NF-κB proteins constitute a group of transcription factors that hold significant significance in the domains of inflammation and immunity [76]. Additionally, NF-κB assumes a pivotal role in diverse processes encompassing development, cellular growth and survival, and proliferation, and its involvement extends to various pathological conditions. Research has demonstrated a strong association between the pathogenesis of HLD and the inflammatory response [77]. Significantly elevated levels of inflammatory cytokines have been observed in the plasma of HLD patients, exerting an impact on their clinical manifestations [78]. Animal experimentation has further revealed the accumulation of copper in the striatum of TX-j mice, accompanied by an increase in inflammatory markers within the striatum and corpus callosum. Consequently, alterations in synaptosomes, particularly in numerous synaptic proteins, occur, thereby influencing motor symptoms [79].

Although there is limited understanding of the precise roles of the majority of DE-lncRNAs, the examination of associated mRNA, GO, and KEGG biological functions reveals a significant correlation with the pathogenesis of HLD and its clinical manifestations. The utilization of bioinformatics analysis offers crucial support for forthcoming investigations on HLD. Furthermore, our findings elucidate the fundamental molecular pathways contributing to the etiology of neurological symptoms in HLD, thereby establishing a basis for its clinical management.

## Conclusions

In conclusion, employing a comprehensive approach involving the integration of RNA-Seq, RT-qPCR, and computational biology, we successfully identified the four lncRNAs (TCONS_00020683, XR_865512.2, XR_001780581.1, and ENSMUST00000207119). Additionally, we established lncRNA-miRNA-mRNA regulatory networks which contribute to explore underlying pathogenesis and therapeutic strategies of HLD. These findings have the potential to serve as valuable biomarkers for the diagnosis and treatment of HLD.

### Supplementary information

ESM 1 (DOCX 26.9 KB)

## Data Availability

The datasets generated for this study can be found in the Sequence Read Archive (https://www.ncbi.nlm.nih.gov/sra) at NCBI, with the BioProject ID: PRJNA799162.
